# Community health workers can improve child growth of antenatally-depressed, South African mothers: a cluster randomized controlled trial

**DOI:** 10.1186/s12888-015-0606-7

**Published:** 2015-09-23

**Authors:** Mark Tomlinson, Mary Jane Rotheram-Borus, Jessica Harwood, Ingrid M. le Roux, Mary O’Connor, Carol Worthman

**Affiliations:** Department of Psychology, Stellenbosch University, Private Bag X1, Matieland, South Africa; Department of Psychiatry and Biobehavioral Sciences, University of California, Los Angeles, USA; Philani Maternal, Child Health, and Nutrition Project, Cape Town, South Africa; Department of Anthropology, Emory University, Atlanta, USA

**Keywords:** Maternal depression, infant growth, stunting, South Africa, community health workers

## Abstract

**Background:**

Maternal antenatal depression has long-term consequences for children’s health. We examined if home visits by community health workers (CHW) can improve growth outcomes for children of mothers who are antenatally depressed.

**Methods:**

A cluster randomized controlled trial of all pregnant, neighbourhood women in Cape Town, South Africa. Almost all pregnant women (98 %, *N* = 1238) were recruited and assessed during pregnancy, two weeks post-birth (92 %) and 6 months post-birth (88 %). Pregnant women were randomized to either: 1) Standard Care (SC), which provided routine antenatal care; or 2) an intervention, The Philani Intervention Program (PIP), which included SC and home visits by CHW trained as generalists (M = 11 visits). Child standardized weight, length, and weight by length over 6 months based on maternal antenatal depression and intervention condition.

**Results:**

Depressed mood was similar across the PIP and SC conditions both antenatally (16.5 % rate) and at 6 months (16.7 %). The infants of depressed pregnant women in the PIP group were similar in height (height-for-age Z scores) to the children of non-depressed mothers in both the PIP and the SC conditions, but significantly taller at 6 months of age than the infants of pregnant depressed mothers in the SC condition. The intervention did not moderate children’s growth. Depressed SC mothers tended to have infants less than two standard deviations in height on the World Health Organization’s norms at two weeks post-birth compared to infants of depressed PIP mothers and non-depressed mothers in both conditions.

**Conclusions:**

A generalist, CHW-delivered home visiting program improved infant growth, even when mothers’ depression was not reduced. Focusing on maternal caretaking of infants, even when mothers are depressed, is critical in future interventions.

**Trial registration:**

ClinicalTrials.gov registration # NCT00996528. October 15, 2009

## Background

In the most recent analyses of the Global Burden of Disease by the World Health Organization, mental and behavioural disorders are the largest contributors to the years lived with disability (YLD), accounting for 22.7 % of all YLD [1]. Depression is the most common of the mental health challenges [2] with the highest YLD (3.7 %), and is about twice as common among women compared to men [3]. Yet, to date, mental illness has not achieved commensurate visibility or funding compared to other diseases, particularly in low and middle income countries (LMIC) [4]. Despite the high prevalence of mental disorders and the known association with poverty [5], data on the consequences of depression, particularly maternal depression, is limited for LMIC [6].

Maternal depression has repeatedly been demonstrated to negatively impact both mothers and their children [7, 8]. Globally, children of depressed mothers show deficits in cognitive, physical, and social development, with the most significant deficits demonstrated among children in LMIC [9]. Children of depressed mothers consistently demonstrate mental health problems [10], yet the infant developmental outcome most affected by maternal depression remains unclear [11, 12].

This study focuses on infants of antenatally depressed mothers in a community sample of pregnant women in South Africa over the first six months of life. Though less well documented than postpartum depression, depression in pregnancy is significantly associated with adverse child outcomes [13–17], including poor child growth [18, 19]. However, depression also co-varies with a range of related stressors. Perinatal depressed mood is also associated with poverty and life stress [20] which are in themselves strongly related to poor child growth. In South Africa, up to 47 % of women have been diagnosed with major depression across the perinatal period [21–23]. Key aspects of infant development (e.g., growth) are compromised in the context of parental poverty and mental health problems [9, 24]. Concurrently, depressed mothers in LMIC are likely to experience overcrowding, food insecurity and poor sanitation [25]. Infant growth is more complex than being based solely on food security and caloric intake. Factors such as the health environment, availability of health care, and feeding and care practices of the mother influence infants’ growth.

In the context of antenatal depression and poverty, retarded infant growth has consistently been linked to maternal depression in Asia, with multiple studies in Pakistan [26] and Vietnam [27]. In Asia, the studies have been conducted with both clinical and community samples, and cross-sectional and longitudinal data. However, there have been inconsistent results regarding the impact of maternal depression on growth in South Africa [28], Ethiopia [27] and Malawi [29].

Identifying sustainable and effective strategies to improve maternal and child outcomes in LMIC is a high priority [30]. Intervention trials with postnatally depressed mothers have hypothesized very different mechanisms by which children are influenced by maternal depression [31]. Depressed mothers may provide sub-optimal care which has detrimental effects on the health of her child [6]. Depression may compromise care-giving behaviours (e.g., ensuring adequate hygiene, optimal nutrition through breastfeeding, immunization, recognizing illness and seeking care), as well as responsive parenting that is needed for physical and mental development of a child. In Pakistan the children of antenatally depressed mothers showed growth retardation in the first year of life [25]. When the mothers received home visits by CHW, maternal depression was reduced, but infants did not improve weight- or length-for-age over the first year of life [32].

Maternal and child health intervention strategies have tended to address only one health risk at a time. As a result many studies provide little insight on the confluence of challenges faced by mothers in LMIC. Our project evaluated a community-based, home visiting program utilising CHW who were trained to address multiple health challenges facing pregnant South African mothers and their newborn infants. The CHW were trained to conduct home visits that addressed nutrition, alcohol, and HIV among pregnant township women, but not maternal depression. The training approach aimed to teach principles of behaviour change [33], based on cognitive-behavioral principles (CBT). CHW supported the mother to problem solve her own situation, rather than to provide solutions for her daily stressors and health challenges. While the CHW did not focus specifically on depressed mood, many of the factors related to positive child outcomes were addressed by the CHW. We report the child growth outcomes over the first six months of life, based on intervention status and maternal depression.

## Methods

The Institutional Review Boards of University of California Los Angeles (UCLA), Stellenbosch University, and Emory University approved the study, whose methods have previously been published [34]. Three independent teams conducted the assessment (Stellenbosch), intervention (Philani Maternal, Child Health, and Nutrition Project, hereafter referred to as Philani), and data analyses (UCLA).

### Study context

Cape Town contains five major peri-urban settlements (townships) with formal and informal rudimentary housing. Unemployment in Cape Town townships is estimated at between 25 and 50 % [35]. Most women live within 5 km of a prenatal clinic. In each area, there is formal housing and vast areas of informal houses (shacks).

### Participants

**Fig. 1:**
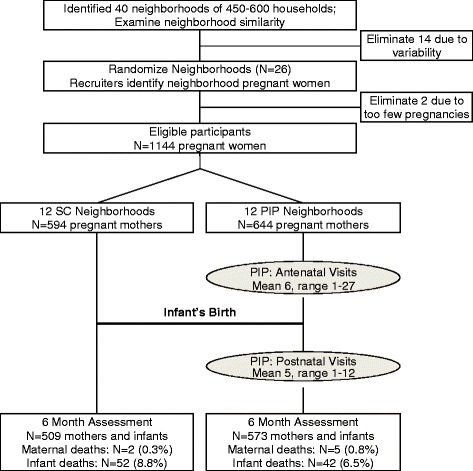
Trial profile

In 2009, 26 township neighborhoods were matched on size (450–600 households), density, public utilities (water, electricity, toilets), distance of primary health care, and the number of alcohol bars. UCLA randomized matched pairs of neighborhoods to either the PIP or SC condition. The minimum number of pregnant women needed per neighbourhood to achieve 80 % power to detect a standardized effect size of 0.40 set the sample size; the original size was 1238. Participant flow through each phase of the study can be seen in Fig. 1.

### Procedure

A recruiter obtained consent to contact and repeatedly visit all households in all neighbourhoods from May 2009 to September 2010 in order to identify all pregnant women. Only 2 % of pregnant women refused participation.

### Intervention conditions

#### Standard Care (SC) condition

All mothers had relatively easy access to antenatal and postnatal care. Treatment for depression is theoretically possible, but it is highly unlikely that health care providers would identify or refer cases of depression, unless symptoms of active psychosis were detected during a clinic visit. Because the HIV rate in this population is about 26 %, standard clinic care includes HIV testing of virtually all women and extensive treatment for Mothers Living with HIV (MLHIV), including the following: dual therapy for Prevent Mother To Child Transmission (PMTCT), referral to antiretroviral therapy (ART) for women with CD4 counts below 200 or World Health Organization Stage 4 disease, the return of PCR test results for infants by 6 weeks of age, and co-trimoxazole for exposed infants starting at 6 weeks of age [36]. Nutritional supplements were also available from clinics during this study period.

#### Philani Intervention Program (PIP)

Philani is a non-governmental organization (NGO) that has been operating in Cape Town townships for more than 30 years. Local township women with good social skills and who were mothers themselves were recruited and trained as CHW to visit the homes of pregnant women and intervene to reduce alcohol misuse, increase adherence to perinatal HIV regimens, and boost child nutrition. CHW’s were trained in: 1) cognitive-behavioural approaches to establishing healthy routines and to problem-solving around goal setting, choices, triggers, and shaping of desirable behaviours; 2) key information about general maternal and child health, techniques for framing each health issue that is a risk (nutrition, alcohol, and HIV), and strategies for applying the health information in families’ daily lives; and 3) coping with their own life challenges. Trainers actively rehearsed and observed videotapes about how to solve challenging life situations, build engaging relationships, and keep responsibility for change with the mother. CHW were supervised weekly (face-to-face supervision as well as via mobile phones), and randomly observed twice a month. Specific PIP content has been described in more detail elsewhere [34].

### Data collection

A driver transported all participants to a central assessment site, allowing interviewers to be blinded to condition. All women provided written informed consent to participate in data collection. All data were collected using mobile phones [37]. Data collectors were taught methods of assessing infants’ growth, strategies for building rapport and collecting honest answers, and how to interact in a non-judgmental manner. Before going into the field, all data collectors observed five client interviews and practiced interviewing skills. All CHW were certified by supervisors prior to becoming interviewers.

### Measures

#### Maternal mood

Maternal mood was assessed at the baseline recruitment interview during pregnancy and at 6 months post-birth using the Edinburgh Postnatal Depression Scale (EPDS), a 10-item measure, with items rated on a scale of 0–3 for severity [38]. The EPDS is a screening tool and is not used to make a confirmatory diagnosis of clinical depression; however, for the purposes of this study, we used a cut-off score of >18 to indicate the probable presence of depression [23]. Screening tools, by definition, will not have the sensitivity or specificity of a clinically validated tool such as the Standard Clinical Interview [39]. If the goal was to measure prevalence, we would have wanted to maximize sensitivity and accept some degree of false-positives. In this study however, we wanted to maximize specificity to reduce false positives, so we used the highest recommended cut-point to increase the chances that a screen positive was a definite case, likely to represent cases of severe depression [40].

#### Infant measures

##### Anthropometric measures

Weight and length was measured at two weeks post birth and at six-months post-birth. Weight was assessed on scales calibrated monthly. Length was measured supine, using a roller meter with infant feet at zero position and bringing the headboard down to the crown of the head. Standardized z-scores (height-for-age, weight-for-age, height-for-weight-for age) were generated using new World Health Organization (WHO) child growth standards for infants under 24 months of age and calculated from WHO Anthro-2005 software. Infants were then categorized as underweight if they had a weight-for-age z-score (WAZ) of < −2 and stunted if they had a length-for-age z-score (HAZ) of < −2 [41]. Newborn infants were also provided with a “Road to Health” card on which birth weight, length at birth and delivery date were recorded. These measures were highly consistent with the interviewers’ measure (r >0.9) and, therefore, we only examine the results using the interviewer collected data.

### Data analysis

Before analyzing infant outcomes, we checked for confounding variables and found no significant differences in baseline demographic characteristics within or across study conditions among the full sample or among mothers depressed antenatally. Using SAS PROC MIXED (version 9°2; SAS Institute Inc., Cary, North Carolina, USA), we modelled infant growth z-scores longitudinally using an unstructured time trend in hierarchical linear regressions that included two random effects: one to model the correlation of repeated measures within a participant and another to model the correlation between participants clustered within the same neighborhood. Explanatory variables included indicators for intervention (PIP; 1 = intervention, 0 = SC) and antenatal depression (DEP; 1 = depressed, 0 = not depressed), time (TIME; 0 = birth, 1 = post-birth, 2 = six months), and the two- and three-way interactions of PIP, DEP, and TIME. Intervention as a moderator of the depression effect on the change in z-score over time (PIP*DEP*TIME) was the effect of interest, and we considered a 2-sided p-value < 0.05 to be significant. Using SAS PROC GLIMMIX, we also modelled the moderating effect of intervention on infant growth (z-score ≥ −2) cross-sectionally at each assessment period using logistic random effects regressions controlling for neighbourhood clustering. Explanatory variables included indicators for intervention (PIP; 1 = intervention, 0 = SC), antenatal depression (DEP; 1 = depressed, 0 = not depressed, and their interaction. Intervention’s moderation of depression’s impact on infant growth (PIP*DEP) was the effect of interest, and we set the criteria for a 2-sided p-value <0.05 to be significant.

## Results

### Sample characteristics

Table 1 summarises the self-reports of mothers in the PIP and SC conditions at the baseline interview conducted at mean of 26 weeks gestational age (SD = 8.2 weeks). There were no differences in gestational age at recruitment across conditions. Women were similar across conditions on: alcohol use in pregnancy (26 %), rates of prior LBW infants (16 %), depression (35 %) and social support measures. They were also highly similar on criteria used in cluster matching such as type of housing (31 % formal), source of water (53 % on site), presence of flush toilet on premises (55 %), and presence of electricity on premises (90 %); and also on demographic characteristics of age, marital status, education, employment status and household income. One significant baseline difference between PIP and SC was noted: among women who had been pregnant before, mothers in the SC had a higher mean number of previous births.

**Table 1:** Baseline characteristics

		intervention (*N* = 644)		SC (*N* = 594)		Total (*N* = 1238)		*P*-Value^*^
Demographic characteristics		*n*	(%)	*n*	(%)	*n*	(%)	
	Mean age (SD)	26.5	(5.5)	26.3	(5.6)	26.4	(5.5)	0.783
	Mean highest education level (SD)	10.3	(1.8)	10.3	(1.8)	10.3	(1.8)	0.639
	Married or lives with partner	377	(58.5)	324	(54.6)	701	(56.6)	0.524
	Sexual partner, past 3 months	580	(90.1)	522	(87.9)	1102	(89.0)	0.284
	Ever employed	129	(20.0)	104	(17.5)	233	(18.8)	0.341
	Monthly household income >2000 Rand	280	(45.6)	279	(48.1)	559	(46.8)	0.484
	Formal housing	197	(30.6)	191	(32.2)	388	(31.3)	0.958
	Water on site	333	(51.7)	327	(55.1)	660	(53.3)	0.983
	Flush toilet	340	(52.8)	343	(57.7)	683	(55.2)	0.923
	Electricity	569	(88.4)	543	(91.4)	1112	(89.8)	0.843
Maternal Health								
	Non-primipara	422	(65.5)	394	(66.3)	816	(65.9)	0.714
	Mean number of live births (SD)	1.5	(0.9)	1.7	(1.1)	1.6	(1.0)	0.005
	Previous LBW infants	61	(14.5)	69	(17.5)	130	(15.9)	0.117
	Tested for TB, lifetime	206	(32.0)	210	(35.4)	416	(33.6)	0.225
	Test positive TB, lifetime	53	(8.2)	50	(9.4)	103	(8.8)	0.438
Mental Health								
	EPDS > 13	238	(37.0)	195	(32.8)	433	(35.0)	0.265
HIV								
	Ever tested for HIV	590	(91.6)	550	(92.6)	1140	(92.1)	0.566
	Women living with HIV	149	(25.5)	146	(26.7)	295	(26.1)	0.649
Alcohol								
	Drank any alcohol, month prior to pregnancy discovery	155	(24.1)	129	(25.8)	284	(24.8)	0.592
	AUDIT-C > 2, month prior to pregnancy discovery	113	(17.6)	101	(20.2)	214	(18.7)	0.323
	Drank any alcohol after pregnancy discovery	56	(8.7)	49	(9.8)	105	(9.2)	0.540
	AUDIT-C > 2, after pregnancy discovery	41	(6.4)	24	(4.8)	65	(5.7)	0.385
	Drank any alcohol, anytime during pregnancy	172	(26.7)	154	(25.9)	326	(26.3)	0.808

^*^P-values from linear (continuous variables) or logistic (binary) random effects regressions, adjusted for neighbourhood clustering

Figure 1 outlines the flow of participants throughout the study. Sample retention rates at follow-up were high, collected within narrow time frames, and similar across intervention conditions: 88 % of participants were assessed at six months (M = 6°8 months, SD = 0°8). There were no significant selection effects between mothers who were successfully reassessed over the six months post-birth and those who were not, and there were no serious adverse events or unintended effects of the study implementation.

As we have reported elsewhere, the intervention did not have an impact on reducing maternal depression at 6 months post-natally [42]. About one in four mothers (30 %) reported depression with a score on the EPDS over 13 and 16.5 % had an EPDS score greater than 18.

**Fig. 2:**
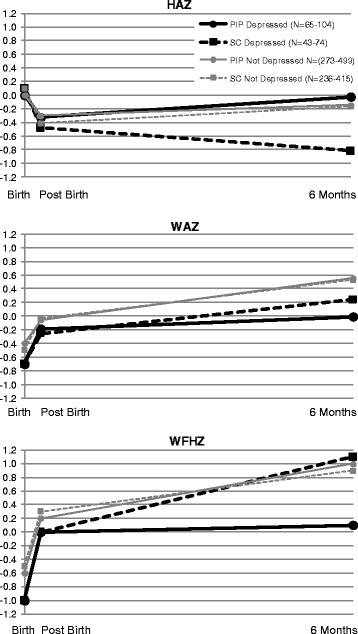
Effect of Philani Intervention program on infant growth

As shown in Fig. 2, the intervention has a significant positive impact on depression’s effect on the change in HAZ between birth and 6 months (estimated mean difference of 0.699; 95 % CI = [0.051, 1.346]; *p* = 0.034). The infants of depressed pregnant women in the PIP group were similar in height to the children of non-depressed mothers in both the PIP and the SC conditions at birth, but significantly taller (HAZ) at 6 months than the infants of pregnant depressed mothers in the SC condition.

WAZ scores were not significantly different between the conditions (PIP vs SC) or depression status at 6 months. Children in each condition and, regardless of maternal depression had WAZ scores above 0 (Z = mean of children globally in WAZ).

There was a significant difference for intervention condition by antenatal status on the change in WFHZ scores between post-birth and 6 months (estimated mean difference of −1.022; 95 % CI = [−1.762, −0.282]; *p* = 0.007). Children of depressed PIP mothers were at the WHO recommended WFHZ at 6 months (i.e., WFHZ = 0); however, the children of the non-depressed mothers in the SC and PIP and children of depressed SC were significantly above the WHO recommended WFHZ at 6 months.

As shown in Table 2, the intervention did not significantly moderate antenatal depression’s effect on infant growth at 6 months. At two weeks post-birth, there was a trend toward the intervention moderating maternal depression’s harmful effect on infant HAZ z ≥ −2, with percentage of infants with HAZ z ≥ −2 higher for depressed mothers compared to non-depressed mothers in the PIP condition (10.8 % vs. 6.7 %, respectively), but lower for depressed vs. non-depressed mothers in the SC group (15.2 % vs. 12.1 %, respectively; *p* = 0.075).

**Table 2:** Intervention’s moderation of depression’s (EPDS > 18) effect on infant growth results at each assessment

		PIP (*N* = 644)										
		EPDS ≤ 18 (*n* = 535)		EPDS > 18 (*n* = 109)		EPDS ≤ 18 (*n* = 499)		EPDS > 18 (*n* = 95)		Depressed-not, PIP vs. SC	OR, PIP vs. SC moderation of depression (95 % CI)*	*P*-Value*
		*n*	%^A^	*n*	%^B^	*n*	%^C^	*n*	%^D^	(B-A)-(D-C)		
Weight-for-age-z-score ≥ −2												
	2 weeks	465	93.2	96	92.3	367	93.1	65	90.3	1.90 %	1.33 (0.51, 3.42)	0.559
	6 months	447	97.8	94	97.9	405	98.1	70	94.6	3.60 %	3.37 (0.54, 21.24)	0.195
Height-for-age-z-score ≥ −2												
	2 weeks	438	89.2	97	93.3	342	87.9	60	84.5	7.50 %	2.34 (0.92, 5.94)	0.075
	6 months	410	91.1	86	89.6	357	87.1	57	77	8.60 %	1.81 (0.76, 4.31)	0.179
Weight-for-height-z-score ≥ −2												
	2 weeks	424	88.9	91	90.1	340	90.7	58	87.9	4.00 %	1.55 (0.56, 4.26)	0.4
	6 months	438	96.5	88	91.7	399	97.3	71	95.9	−3.40 %	0.62 (0.14, 2.76)	0.562

*Results from logistic random effects regression, adjusting for neighborhood clustering; effect of interest is the interaction of PIP and EPDS > 18. ^A^, ^B^, ^C^ and ^D^ equal the percentage of participants from each sub-group with a growth Z-score ≥ -2 at a given time point. The result of (B-A)-(D-C) yields the value below for each row

## Discussion

The children of antenatally depressed mothers in the intervention condition were more often in the normative range for length for their age over time compared to children of depressed mothers in the control condition. Children of antenatally depressed children in the control condition tended to be significantly shorter at birth. Because only 16.5 % were significantly depressed (EPDS < 18), the power to detect significant differences in growth is substantially reduced. The rate of stunting (being two SD lower in height) was more than double at two weeks post birth (OR = 2.36) and similar 6 months later (OR = 1.8) compared to children of depressed mothers in the intervention condition. However, with clustering of neighbourhoods and the reduced sample size, the confidence intervals are too large to have precision in evaluating if this is a significant difference. These rates suggest that CHW may be able to reduce stunting of depressed mothers by focusing on encouraging depressed mothers to maintain caretaking. Our findings contribute to a growing body of literature examining the impact of community based interventions on child health in the context of perinatal depression [32, 43].

The intervention’s negative effect on WFHZ score is a reflection of the positive intervention effect on height (HAZ). Intervention children are taller and significantly less likely to be stunted, their weight for height is at the desirable mean score of 0 (normative weight) at 6 months post birth. Children of antenatally depressed mothers in the control condition were shorter, but of similar weight compared to the children of antenatally depressed mothers in the intervention condition and children of non-depressed mothers in both conditions. Therefore, when calculating WFHZ, the children are relatively heavier at their height, above the desirable mean of 0 and similar to the children of non-depressed mothers in both conditions.

The PIP intervention’s benefit in height was achieved in the context of a study where CHW were not specifically trained to address depression (through screening or treatment) and in which the prevalence of depressive symptoms were highly similar in the intervention and control conditions over time. A study by Rahman and colleagues [32] examined whether or not delivering a cognitive behavioural therapeutic intervention to depressed mothers using village CHW women would improve infant growth [32], and found that directly addressing depression had a large impact on reducing maternal depression, but had no impact on improving infant growth. The work of Tripathy and colleagues in Nepal, on the other hand [43] found significant reductions in neonatal mortality amongst moderately depressed women in a group- based intervention programme aimed at improving social support and problem solving [43]. Similar to findings of our South African study, this result was achieved without specifically targeting perinatal depression. It is likely that the psycho-educational activities of the PIP intervention providing skills to address alcohol, nutrition, and HIV enabled women to be better caretakers of their infants, in spite of being depressed.

Increasingly, antenatal depression is being seen as an important public health concern. The link between postnatal depression and compromised child development is well established [20, 44], but this is less the case with antenatal depression although this relationships is increasingly a target of investigation. Our data suggest that interventions during the antenatal period are crucial. Our findings provide support for training CHW as generalists, in order to address the highest priority health issues in a local community. Training of CHW to address caretaking, problem solving and support, appears to benefit child growth [41]. Treating depression does not necessarily remove the conditions that frequently cause or exacerbate depression – such as poverty and interpersonal violence. On the other hand, providing mothers the tools to help their infants thrive (something most mothers are highly motivated about) allows them to act toward a powerfully motivated goal. Indeed, an inability to help their child thrive may be a real driver of maternal depression. While our findings also provide evidence that there are benefits for child development in the context of perinatal depression, the primary preventive nature of community -based interventions, such as PIP, may not ameliorate moderate to severe depression.

Intervening in multiple health risks concurrently, even by paraprofessional CHW, appears to benefit child growth, even when mothers are depressed during pregnancy and remain depressed. The mechanism by which the caretaking influences height and stunting, but not weight gain is a mystery. It is surprising that the children of PIP mothers who were antenatally depressed had children who were less stunted but did not weigh as much as the children in the SC condition who had depressed mothers. In other analyses we have conducted [40], PIP mothers were significantly more likely to breastfeed their infants and to breastfeed longer. The children of antenatally depressed PIP mothers also breastfed their children longer. Breastfeeding often results in lower weights, which may explain that the intervention children were of normal weight per height.

More research is needed in order to establish the optimal mix of interventions that are sufficiently targeted (screening for perinatal depression and providing referral or treatment), while also being sufficiently generalised and horizontally integrated given the resources constraints of LMIC. Given the explanatory models of mental illness in many countries [11, 45] which link disturbance to social adversity and social problems rather than individualistic explanations, interventions targeted at these social determinants of health may be more acceptable to local communities. Given what is known about the causes of depression, it also may be more appropriate. As a clinical condition, depression is often highly stigmatized as an individual deficiency. In some contexts, interventions may be more efficacious when framed as addressing community challenges, rather than as a treatment program for individuals [11, 45]. The PIP model did not target individual depression, but aimed to improve parenting and child health. Every household was visited in the neighbourhood, regardless of such risks as HIV and alcohol use. The strategic approach adopted in the PIP program is similar to the “Thinking Healthy” programme of Rahman and colleagues [32] and uses cognitive behavioural change principles as a generic method of intervention for a broad range of social problems, rather than depression alone.

The relationship between maternal mental health and child growth is complex [12]. It is possible that interventions focussed on maternal depression may not fully address this complexity [46], while generalist models of implementation are not sufficiently targeted to make a significant impact on moderate to severe perinatal maternal depression. Evidence is needed on what the optimal implementation models are. The improvements we have seen in this study warrant continued tracking to examine sustainability and durability of an intervention’s impact when delivered by CHW.

Finally, Philani is a NGO which has survived 30 years. Over that time, it has developed core relationships with staff in the Department of Health, community leaders, church officials, clinics and a broad range of key community roles. Unlike many other NGOs, it has well-developed accountability and training systems for both CHW and supervisors. Diffusion of this model to other NGO without these strengths is likely to face many obstacles. The strengths of this study are that it is a cluster randomized controlled trial of mothers monitored during pregnancy and the similarity of the mothers and infants and the high retention in the intervention and the control condition are additional evidence of the strengths of this design. We did not conduct full diagnostic interviews for depression, which is a limitation of our current design.

## Conclusion

We showed benefits to infant growth in the context of antenatal depression when community health workers intervened to address concurrent community health risks, but not specifically trained to address depression through screening or treatment. This adds to a growing body of evidence identifying sustainable and effective strategies to improve maternal and child outcomes in LMIC.
